# The Presence of Microplastics in Human Semen and Their Associations with Semen Quality

**DOI:** 10.3390/toxics13070566

**Published:** 2025-07-03

**Authors:** Yi Guo, Mengxun Rong, Yuping Fan, Xiaoming Teng, Liping Jin, Yan Zhao

**Affiliations:** 1 Center for Reproductive Medicine, Shanghai First Maternity and Infant Hospital, School of Medicine, Tongji University, Shanghai 200092, China; gy_guoyi@tongji.edu.cn (Y.G.); fanyuping@51mch.com (Y.F.); tengxiaoming@51mch.com (X.T.); 2 School of Public Health, Key Laboratory of Public Health Safety of Ministry of Education, Fudan University, Shanghai 200032, China; 23211020089@m.fudan.edu.cn; 3 Center of Big Data & Biobank, Hospital of Obstetrics and Gynecology, Shanghai Medical School, Fudan University, Shanghai 200090, China

**Keywords:** microplastics, semen quality, reproductive toxicity, sperm motility

## Abstract

Microplastics (MPs) are becoming one of the most serious environmental threats worldwide. They have been shown to induce male reproductive toxicity in animal studies. However, evidence of their adverse effects on male reproductive health in human is still lacking. In this study, we evaluated the presence of MPs in human semen and explored their associations with semen quality. A total of 45 semen samples from men attending a fertility center were collected. MPs in the semen samples were analyzed by laser direct infrared (LD-IR) spectroscopy. MPs were found in 34 out of 45 semen samples, with an average abundance of 17.0 (42.0) particles/g. The size of MPs ranged from 20.3 μm to 189.7 μm and the majority (57.8%) were smaller than 50 μm. A total of 15 distinct MPs polymers were identified, and polyethylene (PET) accounted for 35.9% of the total amount of MPs, followed by butadiene rubber (BR, 26.4%) and chlorinated polyethylene (CPE, 12.2%). Analysis of the association of MP exposure with semen quality showed that participants exposed to PET MPs experienced a reduction in sperm progressive motility (20.6% ± 12.8% vs. 34.9% ± 15.9%, *p* = 0.056). However, no significant association was found between MP exposure and sperm concentration or total sperm count. Our findings confirmed the presence of MPs in human semen and suggested that MP exposure might have adverse impacts on male reproductive health. However, further large-scale studies are needed to confirm these findings.

## 1. Introduction

Microplastics (MPs), which are defined as plastic particles or fibers smaller than 5 mm in size [1], have been frequently detected in terrestrial and aquatic ecosystems [2,3,4]. Moreover, MPs have also been found in various foods, bottled water, tap water and salt [4,5]. Because of the increased use of plastic products and their persistent nature, MPs are becoming one of the most serious environmental threats worldwide.

Human exposure to MPs mainly occurs through ingestion of contaminated food and water, inhalation of air and direct dermal contact [6,7]. Several recent studies have reported the presence of MPs in different human biological samples such as blood [8], colectomy specimens [9], saliva [10], sputum [11], lung [12], liver [13], placenta [14,15], breastmilk [14,16] and feces [17,18,19], which suggests that MPs could distribute throughout the human body and accumulate in some organs. Thus, there have been considerable concerns about their potential adverse effects on human health.

The human reproductive system is highly sensitive to various environmental stresses [20,21]. MPs, especially polystyrene MPs (PS MPs), have been shown to induce male reproductive toxicity in various organisms [22,23,24,25]. Recent studies on mice demonstrated that PS MP exposure resulted in significant decreases in sperm number and motility and induced abnormal sperm morphology, such as acrosome loss, no head, small head, cervical folding and tail loss [26,27]. However, limited research has been conducted on humans.

In a pilot study conducted in Beijing, Zhao et al. first detected six types of MP polymers in human testis and semen [28]. Another study reported the presence of eight types of MP polymers in 10 human semen samples collected from a polluted area in Southern Italy [29]. This evidence suggested the presence of MPs in the human male reproductive system. However, only two recent studies have investigated the associations of MP exposure with semen quality [30,31]. Therefore, in this present study, we aimed to detect MPs in semen samples of men attending a fertility center and explore the potential associations of MP exposure with semen quality.

## 2. Materials and Methods

### 2.1. Study Participants

This study has been reviewed and approved by the Human Ethical Committee of the Shanghai First Maternity and Infant Hospital (No. KS23198). Participants were male patients who visited the hospital’s Reproductive Medical Center seeking assisted reproductive technology (ART) procedures between 14 February 2023 and 16 May 2023. A total of 45 patients who were less than 40 years old and had no chronic diseases (diabetes, hypertension, liver, kidney, or thyroid diseases) were included. They all signed informed consent prior to participating. All participants were interviewed by trained physicians. The basic demographic characteristics collected included ethnicity, age, body weight and height, income and educational level, alcohol consumption and cigarette smoking.

### 2.2. Semen Collection and Quality Analysis

Semen samples were collected by masturbation. To ensure that no contamination occurred during sample collection, each participant was provided with a glass container. Each semen sample was divided into two parts: one was sealed in cooled containers and used for MPs analysis; the other part was liquefied in a 37 °C heating chamber and used for semen quality analysis.

The semen parameters of sperm count and motility were evaluated through a computer-assisted semen analysis (CASA) system. Sperm motility comprised progressive motility, non-progressive motility and immotile spermatozoa. Progressive motility was the percentage of spermatozoa with rapid and slow progressive motility, while non-progressive motility was the percentage of spermatozoa with local motility. Followed the World Health Organization (WHO, 2010) guidelines, quality control procedures of semen analysis were established by our semen laboratory and routinely conducted by laboratory technicians [32].

### 2.3. Identification and Quantification of MPs in Human Semen Samples

Semen samples were weighed and digested. The protocol for digestion was adapted from the method of Cole et al. (2014) [33], with small modifications. Specifically, an enzyme liquid system, which consisted of 1 mg/mL proteinase K, 0.05% SDS solution, 1 mol Tris-HCl solution and 5 mmol CaCl_2_ solution, was added to each beaker with a 2–3 g semen sample. Then, these beakers were placed in an incubator at 60 °C for 8 h. After that, the beakers were incubated for 3 days at room temperature. Finally, all solutions were filtered through a stainless steel membrane (10 μm pore size, Shanghai Suyan Sieve Mesh Products Co., Ltd., Shanghai, China). Next, the membrane was placed into a 20 mL glass vial and washed with 15 mL anhydrous ethanol solution (Sinopharm Chemical Reagent Co., Ltd., Shanghai, China). After a 40 KHz ultrasonic treatment (Shenzhen Jiemeng Co., Ltd., Shenzhen, China) for 30 min, all particles on the filter surface had fallen into the solution. Lastly, the obtained ethanol dispersion was placed in an infrared drying oven (Hangzhou Qiwei Instrument Co., Ltd., Hangzhou, China) for concentration until it reached approximately 500 μL. The concentrated solution was then dripped onto the high-reflective glass of the LD-IR using a glass dropper. After the ethanol had completely evaporated, the microplastics deposited onto the slides were prepared for the LD-IR test.

Laser direct infrared spectroscopy (LD-IR) (8700, Agilent Technologies Inc., Santa Clara, CA, USA) was used to identify and quantify MPs in the digested samples. Due to the limitations of LD-IR, only the particles with diameters of 20–500 μm were identified and counted. Using the trans-reflection mode (TRM), we focused on each particle with wavenumbers from 900 to 1800 cm^−1^. Once all suspected particles were located, bundled image analysis software (version 1.4.10, Agilent Technologies Inc., Santa Clara, CA, USA) was used to collect the size, shape and number of MPs. To identify the candidate MPs polymers, the obtained information was matched with the Agilent spectrum libraries. In our study, a degree of match higher than 80% was set as the threshold quality for polymer identification.

### 2.4. Quality Assurance and Quality Control

No plastic device was used during the sample collection, storage, digestion and LD-IR analysis. All glass devices used during the experiment were rinsed with anhydrous ethanol 3 times and cauterized with an open flame before use. All reagents used in the sample digestion and LD-IR analysis were vacuum-filtered through a 0.45 μm stainless steel membrane and detected by LD-IR to ensure that the number of MPs was less than 3 in 5 mL reagent. Along with all semen samples, three procedural blank samples containing anhydrous ethanol were processed and analyzed to monitor background contamination.

### 2.5. Statistical Analysis

The general characteristics and semen parameters were exhibited as means (SDs) for continuous variables and percentages (%) for categorical variables. The abundance (particles/g) of MPs was depicted as means (SDs) and percentiles. The Mann–Whitney U test was applied to compare the differences in semen parameters between exposure and non-exposure groups. For each polymer type with >10% detection frequency, the exposure group was defined as participants with detected MPs of the corresponding polymer in their semen samples, while the non-exposure group was defined as participants with no detected corresponding polymer. SPSS 16.0 was used for all data analysis, with a two-sided *p* value < 0.05 being considered statistically significant.

## 3. Results

### 3.1. Demographic Characteristics of the Participating Men

The general characteristics and semen parameters of the subjects are shown in Table 1. The mean age of the participants was 31.1 ± 3.0 years, with a mean body mass index (BMI) of 24.1 ± 2.9 kg/m^2^. The majority (75.6%) of the participating men had an educational level of college or above. Of the 45 included men, 13 (28.9%) were current cigarette smoker, whereas only 9 (20.0%) reported current alcohol consumption. The mean (SD) sperm concentration was 23.7 (14.7) × 10^6^/mL and the mean total sperm count was 94.0 (7.0) × 10^6^. The mean (SD) progressive, non-progressive sperm motility and immotile spermatozoa were 33.3% (16.1%), 13.9% (6.0%) and 52.8% (17.9%), respectively.

### 3.2. Occurrence and Abundance of MPs in Semen Samples

The detection frequencies of MPs are depicted in Table 2. Of the 45 semen samples, at least one MP was detected in 34 (75.6%). The average abundance of MPs in the 45 semen samples was 17.0 ± 42.0 particles/g with a median of 3.9 particles/g. Regarding microplastic polymers, butadiene rubber (BR) and chlorinated polyethylene (CPE) were the most frequently detected polymers followed by polypropylene (PP), polyethylene terephthalate (PET), fluororubber (Flu) and polyethylene (PE), with detection frequencies of 28.9%, 28.9%, 26.7%, 11.1%, 11.1% and 11.1%, respectively. The average abundances of BR, CPE, PP, PET, Flu and PE were 4.5 (11.6), 2.1 (4.9), 1.2 (3.5), 6.1 (38.8), 0.5 (1.7) and 0.3 (1.0) particles/g, respectively. Table S1 presents the concentrations of various polymer types in detected microplastics from each participant’s semen sample.

### 3.3. Shape and Size Distribution of MPs in Semen Samples

**Figure 1:**
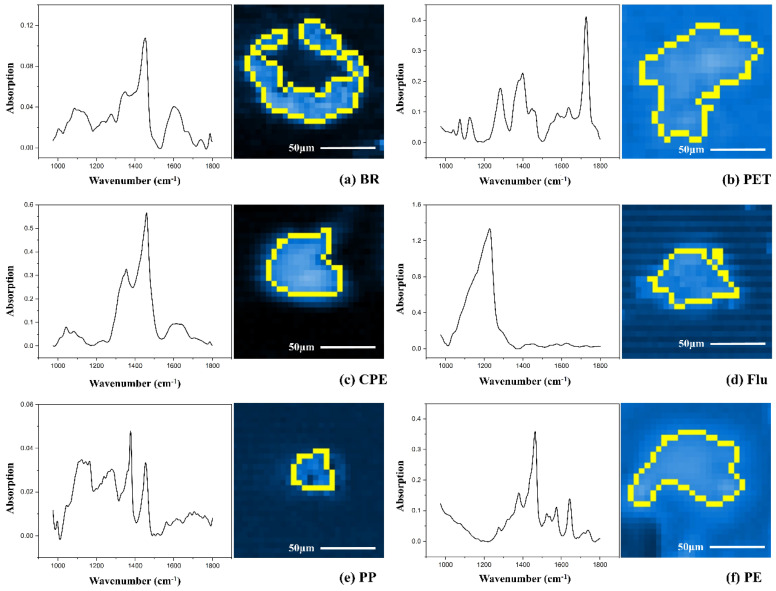
LD-IR scanning images of typical microplastics in semen (yellow outline) and the corresponding infrared spectrum. (**a**) Butadiene rubber, BR; (**b**) polyethylene terephthalate, PET; (**c**) chlorinated polyethylene, CPE; (**d**) fluororubber, Flu; (**e**) polypropylene, PP; (**f**) Polyethylene, PE.

The spectrograms of six typical MPs are shown in Figure 1. Based on the essential parameters, such as diameter, height, width, aspect ratio and area, of the MPs detected in semen samples, MPs were divided into film and fragment. Among the detected MPs, film was the predominant shape, accounting for 63.7%, whereas fragment accounted for 36.3% of the total MPs.

The LD-IR microscopy facilitated the detection of MPs with a diameter ranging from 20 to 500 μm. The average size of all MPs was 53.0 (31.9) μm, ranging from 20.3 μm to 189.7 μm. MPs in semen samples were predominantly 20–50 μm in size (60%), followed by 50–100 μm (33.2%), 100–150 μm (7.4%) and 150–200 μm (1.6%).

### 3.4. Polymer Compositions of Microplastics in Semen Samples

**Figure 2:**
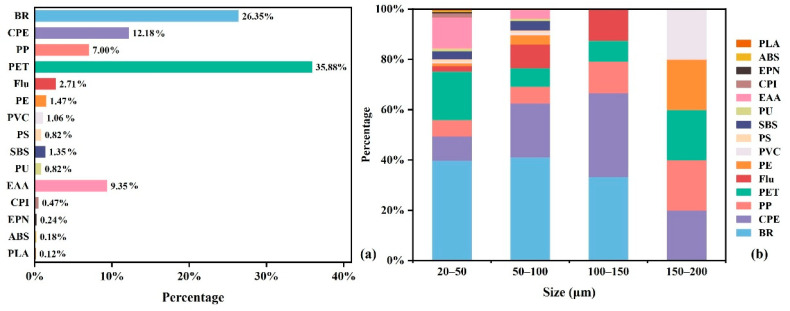
Polymer compositions of MPs in human semen. (**a**) Polymer compositions of all MPs. PET accounted for 35.9% of the total amount of MPs, followed by BR (26.4%) and CPE (12.2%). (**b**) Polymer compositions of different size ranges of MPs. MP polymer types differed by size categories.

The polymer compositions of microplastics are shown in Figure 2a. A total of 15 distinct MP polymers were identified and verified. In all MP particles, PET accounted for 35.9% of the total amount followed by BR (26.4%) and CPE (12.2%). MP polymer types differed by size category. As shown in Figure 2b, BR (39.8%) and PET (19.4%) were the predominant polymers in the size range of 20–50 μm. However, in the size ranges of 50–100 μm and 100–150 μm, BR and CPE were the main MPs polymers. The compositions of MP polymer types within different size ranges is comprehensively presented in Table S2.

### 3.5. Associations Between MP Exposure and Semen Quality

Table 3 shows the differences in semen parameters between exposure and non-exposure groups. Participants exposed to PET MPs showed a trend toward reduced semen progressive motility (yes vs. no: 20.6% ± 12.8% vs. 34.9% ± 15.9%; *p* = 0.056) and a potential increase in immotile spermatozoa (yes vs. no: 66.7% ± 17.5% vs. 51.1% ± 17.4%; *p* = 0.080). However, no association was found between MP exposure and sperm concentration or total sperm count.

## 4. Discussion

MPs are emerging contaminants that are ubiquitous in the environment and in the human body [34]. Given their ability to enter the male reproductive system, the potential impact of MPs on semen quality cannot be ignored. In this study, using LD-IR microscopy, we identified 15 microplastic polymers in 34 out of 45 semen samples. Besides our study, four previous studies have also reported the presence of MPs in human semen. In one study conducted in Beijing, China, using the same detection technology, Zhao et al. identified six polymer types in 11 out of 25 semen samples [28]. In another three studies, eight polymers were detected in semen samples by Raman microspectroscopy [29,30,31]. Those results together indicate that MPs have polluted human semen.

Regarding the morphological characteristics of MPs, we found that MPs in sizes ranging from 20 to 100 μm accounted for more than 80% of all MPs. This observation is consistent with the study conducted in Beijing, China, which also employed LD-IR microscopy. Additionally, we found that PET and BR were the dominant components. However, in the studies conducted in Beijing, China [28], PVC and PE were the most frequent polymers. When it comes to the detection methods of MPs, LD-IR microscopy is more sensitive to changes in polar functional groups, whereas Raman microspectroscopy provides a wider spectral coverage and is more sensitive to nonpolar functional groups. Due to the differences in detection capability, we were unable to compare our result with those that employed Raman microspectroscopy [29,30,31].

Although the presence of MPs in human semen has been confirmed, their adverse effects on semen quality remain unclear. In a single-center study in China, Li et al. revealed that semen exposed to PS demonstrated higher sperm progressive motility as compared to the PVC exposure group [30]. In another multi-site study, the authors demonstrated that polytetrafluoroethylene (PTFE) exposure was significantly associated with reduced sperm count and motility [31]. In the present study, we found that participants exposed to PET MPs showed a reduction in sperm progressive motility and an increase in immotile spermatozoa. Those results together highlight the potential reproductive health risks posed by MP exposure in humans.

The mechanisms by which MP exposure may impair semen quality are not yet fully understood. The blood–testis barrier (BTB) is a distinctively junctional structure, which is crucial for normal spermatogenesis [35]. Studies have shown that MP exposure could disrupt the BTB integrity through reactive oxygen species (ROS)-mediated imbalance of mTOR complex 1 (mTORC1) and mTORC2 [36]. In addition, MP exposure was also reported to have induced a significant decrease in testosterone levels by downregulating the luteinizing hormone (LH)-mediated steroidogenic enzymes and steroidogenic acute regulatory protein (StAR) pathway [22]. We propose that the disruption in BTB integrity and the reduction in testosterone levels induced by MP exposure may partly account for a decline in sperm quality.

In this study, we confirmed the presence of MPs in human semen and observed a potential link between PET MP exposure and sperm motility. However, there are some limitations in our study. Firstly, although the accuracy of LD-IR microscopy in component detection is higher than in other detection methods, it was unable to count MPs with diameters less than 20 μm, which may have led to an underestimation of the exposure levels of MPs in the human semen. Secondly, despite the strict plastic-free procedures that were followed throughout the process of semen sample collection and MP detection, the risk of contamination still cannot be fully ruled out. Thirdly, the sample size of this present study is relatively small and we could not adjust for potential confounders when examining the association of MP exposure and semen quality. Finally, we lack data on the exposure of other environmental or occupational pollutants, which may also impact sperm motility. A more rigorous study design that could control for multiple confounders is needed to confirm our findings.

## 5. Conclusions

Using the LD-IR microscopy, we identified 15 distinct MPs polymers in 34 out of 45 semen samples, which confirmed the presence of MPs in the human semen. Moreover, we found that participants exposed to PET MPs showed a reduction in sperm progressive motility. Our findings suggest that MP exposure might interfere with the male reproductive system and highlight the need to reduce MP exposure in reproductive-aged men. While these preliminary findings suggest potential risks of microplastics to the male reproductive health, further large-scale studies are needed to confirm these observations.

## Figures and Tables

**Table 1:** The general characteristics and semen parameters of the study participants (*N* = 45).

Characteristic	Mean (SD) or N (%)
Age (years)	31.1 (3.0)
BMI (kg/m^2^)	24.1 (2.9)
18.5–23.9	22 (48.9%)
24.0–27.9	18 (40.0%)
≥28.0	5 (11.1%)
Educational level	
Middle school or below	6 (13.3%)
High school	5 (11.1%)
College or above	34 (75.6%)
Current cigarette smoker	13 (28.9%)
Current alcohol consumption	9 (20.0%)
Semen parameters	
Concentration (10^6^/mL)	23.7 (14.7)
Total sperm count (×10^6^)	94.0 (7.0)
Progressive motility (%)	33.3 (16.1)
Non-progressive motility (%)	13.9 (6.0)
Immotile spermatozoa (%)	52.8 (17.9)

Abbreviation: SD, standard deviation; BMI, body mass index.

**Table 2:** The detection rates and descriptive statistics of MPs (particles/g) detected in human semen (*N* = 45).

Categories	Detection Rate (%)	Mean	SD
Total MPs	75.6	17.0	42.0
BR	28.9	4.5	11.7
CPE	28.9	2.1	4.9
PP	26.7	1.2	3.5
PET	11.1	6.1	38.8
Flu	11.1	0.5	1.7
PE	11.1	0.3	1.0
PVC	6.7	0.2	0.8
PS	6.7	0.1	0.7
SBS	4.4	0.2	1.2
PU	4.4	0.1	0.7
EAA	2.2	1.6	10.7
CPI	2.2	0.1	0.6
EPN	2.2	0.0	0.3
ABS	2.2	0.0	0.2
PLA	2.2	0.0	0.1

Abbreviations: BR, butadiene rubber; CPE, chlorinated polyethylene; PP, polypropylene; PET, polyethylene terephthalate; Flu, fluororubber; PE, polyethylene; PVC, polyvinyl chloride; PS, polystyrene; SBS, styrene-butadiene-styrene; PU, polyurethane; EAA, ethylene acrylic acid; CPI, chlorinated polyisoprene; EPN, phenolic epoxy resin; ABS, acrylonitrile butadiene styrene; PLA, polylactic acid.

**Table 3:** The associations between MP exposure and semen parameters.

	Sperm Concentration (10^6^/mL)	Total Sperm Count (×10^6^)	Progressive Motility (%)	Non-Progressive Motility (%)	Immotile Spermatozoa (%)
Total MPs					
No (n = 11)	18.6 ± 12.4	80.7 ± 52.7	34.5 ± 15.7	13.6 ± 5.3	51.9 ± 15.4
Yes (n = 34)	25.3 ± 15.2	98.3 ± 75.4	32.9 ± 16.4	14.0 ± 6.3	53.1 ± 18.9
BR					
No (n = 32)	22.6 ± 14.8	97.7 ± 79.2	31.5 ± 16.7	13.4 ± 4.7	55.1 ± 17.0
Yes (n = 13)	26.4 ± 14.7	85.0 ± 43.0	37.6 ± 14.2	15.3 ± 8.5	47.2 ± 19.5
CPE					
No (n = 32)	24.8 ± 15.1	99.6 ± 77.3	33.6 ± 15.4	14.0 ± 4.8	52.4 ± 16.3
Yes (n = 13)	20.9 ± 14.0	80.2 ± 49.5	32.5 ± 18.5	13.6 ± 8.6	53.8 ± 22.1
PP					
No (n = 33)	22.8 ± 14.0	86.5 ± 51.8	31.5 ± 16.2	14.2 ± 6.6	54.3 ± 18.5
Yes (n = 12)	26.2 ± 17.1	114.8 ± 106.6	38.1 ± 15.3	13.2 ± 4.0	48.7 ± 16.2
PET					
No (n = 40)	23.5 ± 14.8	95.6 ± 72.6	34.9 ± 15.9 ^#^	14.1 ± 6.2	51.1 ± 17.4 ^#^
Yes (n = 5)	24.7 ± 16.0	81.1 ± 54.0	20.6 ± 12.8 ^#^	12.8 ± 5.3	66.7 ± 17.5 ^#^
Flu					
No (n = 40)	23.5 ± 15.2	94.4 ± 73.5	32.4 ± 16.5	13.9 ± 6.2	53.7 ± 18.6
Yes (n = 5)	24.9 ± 10.9	91.1 ± 42.2	40.3 ± 11.0	14.3 ± 5.0	45.4 ± 9.1
PE					
No (n = 40)	22.6 ± 14.2	91.1 ± 69.4	33.0 ± 16.6	13.9 ± 6.2	53.1 ± 18.4
Yes (n = 5)	32.6 ± 17.7	117.7 ± 82.2	35.5 ± 13.1	13.8 ± 5.3	50.8 ± 14.8

Abbreviations: BR, butadiene rubber; CPE, chlorinated polyethylene; PP, polypropylene; PET, polyethylene terephthalate; Flu, fluororubber; PE, polyethylene. # *p* < 0.10.

## Data Availability

Data will be made available on request.

## Supplementary Materials

The following supporting information can be downloaded at https://www.mdpi.com/article/10.3390/toxics13070566/s1: Table S1. The descriptive statistics of MPs (particles/g) detected in the semen samples of each participant (*N* = 45); Table S2. The compositions of microplastic polymer types within each size range in the human semen.

## Abbreviations

The following abbreviations are used in this manuscript:

MPs	Microplastics
LD-IR	Laser direct infrared spectroscopy
ART	Assisted reproductive technology
CASA	Computer-assisted semen analysis
WHO	World Health Organization
BMI	Body mass index
BR	Butadiene rubber
CPE	Chlorinated polyethylene
PP	Polypropylene
PET	Polyethylene terephthalate
Flu	Fluororubber
PE	Polyethylene
PS	Polystyrene
