# Supplementary material for: The Presence of Microplastics in Human Semen and Their Associations with Semen Quality
Source: Toxics. 2025 Jul 3;13(7):566. doi: 10.3390/toxics13070566 (PMC12299061; doi:10.3390/toxics13070566)
Supplement: Supplementary file 1 [file toxics-13-00566-s001.zip › toxics-3676914-supplementary.pdf]

Supplementary Material

The presence of microplastics in human semen and their associations with semen quality

Yi Guo [a, †], Mengxun Rong [b, †], Yuping Fan [a], Xiaoming Teng [a, *], Liping Jin [c, *], Yan Zhao [c, *]

[a] Center for Reproductive Medicine, Shanghai First Maternity and Infant Hospital, School of Medicine, Tongji University, Shanghai, China.

[b] School of Public Health, and Key Laboratory of Public Health Safety of Ministry of Education, Fudan University, Shanghai, China

[c] Center of Big Data & Biobank, Hospital of Obstetrics and Gynecology, Shanghai Medical School, Fudan University, Shanghai, China

Author Contributions:

Yi Guo and Mengxun Rong contributed equally to this work.

Corresponding Authors:

Yan Zhao

Center of Big Data & Biobank, Hospital of Obstetrics and Gynecology, Shanghai Medical School, Fudan University, Shanghai, China. Email: zy861201@163.com

Liping Jin

Hospital of Obstetrics and Gynecology, Shanghai Medical School, Fudan University, Shanghai, China. Email: jinlp01@163.com

Xiaoming Teng

Center for Reproductive Medicine, Shanghai First Maternity and Infant Hospital, School of Medicine, Tongji University, Shanghai, China. Email: tengxiaoming@51mch.com

Table S1. The descriptive statistics of MPs (particles/g) detected in semen sample of each participant (N=45).

ID	BR	CPE	PP	PET	Flu	PE	PVC	PS	SBS	PU	EAA	CPI	EPN	ABS	PLA
#01	0.0	4.5	0.0	0.0	0.0	0.0	0.0	0.0	0.0	0.0	0.0	0.0	0.0	0.0	0.0
#02	0.0	0.0	0.0	2.7	0.0	0.0	0.0	0.0	0.0	0.0	0.0	0.0	0.0	0.0	0.0
#03	0.0	0.0	0.0	0.0	0.0	0.0	0.0	0.0	0.0	0.0	0.0	0.0	0.0	0.0	0.0
#04	0.0	0.0	0.0	0.0	0.0	0.0	0.0	0.0	0.0	0.0	0.0	0.0	0.0	0.0	0.0
#05	0.0	5.5	0.0	0.0	0.0	0.0	0.0	0.0	0.0	0.0	0.0	0.0	0.0	0.0	0.0
#06	0.0	0.0	0.0	0.0	0.0	0.0	0.0	0.0	0.0	4.8	0.0	0.0	0.0	0.0	0.0
#07	0.0	0.0	1.0	0.0	0.0	0.0	0.0	0.0	0.0	0.0	0.0	0.0	0.0	0.0	0.0
#08	0.0	0.0	0.0	0.0	0.0	1.9	1.9	0.0	0.0	0.0	0.0	0.0	0.0	0.0	0.0
#09	0.0	0.0	0.0	0.0	0.0	0.0	0.0	0.0	0.0	0.0	0.0	0.0	0.0	0.0	0.0
#10	13.3	0.0	0.0	0.0	0.0	0.0	0.0	0.0	0.0	0.0	0.0	0.0	0.0	0.0	0.0
#11	0.0	0.0	4.7	0.0	0.0	0.0	0.0	0.0	0.0	0.0	0.0	0.0	0.0	0.0	0.0
#12	0.0	0.0	0.0	0.0	0.0	0.0	0.0	0.0	0.0	0.0	0.0	0.0	0.0	0.0	0.0
#13	0.0	0.0	0.0	6.9	0.0	0.0	0.0	0.0	0.0	0.0	0.0	0.0	0.0	0.0	0.0
#14	0.0	0.0	1.5	0.0	0.0	0.0	0.0	0.0	0.0	0.0	0.0	0.0	0.0	0.0	0.0
#15	0.0	0.0	0.0	260.3	0.0	0.0	0.0	0.0	0.0	0.0	0.0	0.0	0.0	0.0	0.0
#16	1.5	0.0	1.5	3.0	0.0	0.0	0.0	0.0	0.0	0.0	0.0	0.0	0.0	0.0	0.0
#17	0.0	0.0	0.0	0.0	0.0	0.0	0.0	0.0	0.0	0.0	0.0	0.0	0.0	0.0	0.0
#18	0.0	0.0	6.4	0.0	0.0	0.0	0.0	0.0	0.0	0.0	0.0	0.0	0.0	0.0	0.0
#19	0.0	0.0	2.4	0.0	0.0	0.0	0.0	0.0	0.0	0.0	0.0	0.0	0.0	0.0	0.0
#20	0.0	0.0	0.0	0.0	0.0	0.0	0.0	0.0	0.0	0.0	0.0	0.0	0.0	0.0	0.0
#21	0.0	0.0	0.0	0.0	0.0	0.0	0.0	0.0	0.0	0.0	0.0	0.0	0.0	0.0	0.0
#22	0.0	0.0	0.0	0.0	0.0	0.0	0.0	0.0	0.0	0.0	71.6	0.0	0.0	0.0	0.0
#23	0.0	20.6	0.0	0.0	0.0	0.0	0.0	0.0	0.0	0.0	0.0	0.0	0.0	0.0	0.0
#24	0.0	0.0	0.0	0.0	0.0	0.0	0.0	0.0	0.0	0.0	0.0	0.0	0.0	0.0	0.0

#25	0.0	0.0	2.3	0.0	6.9	0.0	0.0	0.0	0.0	0.0	0.0	0.0	0.0	0.0	0.0
#26	0.0	0.0	2.5	0.0	0.0	0.0	0.0	1.3	0.0	0.0	0.0	0.0	0.0	0.0	0.0
#27	0.0	0.0	0.0	0.0	0.0	0.0	0.0	0.0	0.0	0.0	0.0	0.0	0.0	0.0	0.0
#28	38.0	12.0	0.0	0.0	8.0	6.0	0.0	0.0	0.0	0.0	0.0	0.0	2.0	0.0	0.0
#29	5.3	5.3	21.3	0.0	0.0	0.0	0.0	0.0	0.0	0.0	0.0	0.0	0.0	0.0	0.0
#30	0.0	0.7	0.0	0.0	0.0	0.0	0.0	0.0	0.0	0.0	0.0	0.0	0.0	0.0	0.0
#31	0.0	1.0	0.0	0.0	0.0	0.0	0.0	0.0	0.0	0.0	0.0	0.0	0.0	0.0	0.0
#32	0.7	1.4	0.0	0.0	0.0	0.0	0.0	0.0	0.0	1.4	0.0	0.0	0.0	0.0	0.7
#33	0.0	0.0	0.0	1.3	0.0	1.3	0.0	0.0	0.0	0.0	0.0	0.0	0.0	0.0	0.0
#34	3.7	1.2	0.0	0.0	0.0	0.0	1.2	0.0	0.0	0.0	0.0	3.7	0.0	0.0	0.0
#35	0.0	0.0	0.0	0.0	0.0	0.0	0.0	0.0	0.0	0.0	0.0	0.0	0.0	0.0	0.0
#36	1.0	0.0	1.0	0.0	1.0	1.0	0.0	0.0	0.0	0.0	0.0	0.0	0.0	0.0	0.0
#37	1.0	0.0	2.0	0.0	0.5	0.0	0.0	0.0	0.0	0.0	0.0	0.0	0.0	0.0	0.0
#38	1.8	0.0	0.0	0.0	0.0	0.0	0.0	0.0	0.0	0.0	0.0	0.0	0.0	0.0	0.0
#39	14.7	3.4	0.0	0.0	0.0	0.0	0.0	0.0	2.7	0.0	0.0	0.0	0.0	0.0	0.0
#40	0.0	0.0	0.0	0.0	0.0	0.0	0.0	0.0	0.0	0.0	0.0	0.0	0.0	0.0	0.0
#41	49.5	19.8	0.0	0.0	0.0	0.0	5.0	0.0	0.0	0.0	0.0	0.0	0.0	0.0	0.0
#42	32.5	13.7	0.0	0.0	0.0	1.3	0.0	0.0	7.5	0.0	0.0	0.0	0.0	1.3	0.0
#43	0.0	0.0	0.0	0.0	0.0	0.0	0.0	4.1	0.0	0.0	0.0	0.0	0.0	0.0	0.0
#44	38.5	4.2	0.0	0.0	0.0	0.0	0.0	1.0	0.0	0.0	0.0	0.0	0.0	0.0	0.0
#45	0.0	0.0	6.8	0.0	4.5	0.0	0.0	0.0	0.0	0.0	0.0	0.0	0.0	0.0	0.0
Total	201.5	93.3	53.4	274.2	20.9	11.5	8.1	6.4	10.2	6.2	71.6	3.7	2	1.3	0.7

Abbreviations: BR, butadiene rubber; CPE, chlorinated polyethylene; PP, polypropylene; PET, polyethylene terephthalate; Flu, fluororubber; PE, polyethylene; PVC, polyvinyl chloride; PS, polystyrene; SBS, styrene-butadiene-styrene; PU, polyurethane; EAA, ethylene acrylic acid; CPI, chlorinated polyisoprene; EPN, phenolic epoxy resin; ABS, acrylonitrile butadiene styrene; PLA, polylactic acid.

Table S2. The compositions of microplastic polymer types within each size range in human semen.

Categories	Size range (μm)			
	**20-50**	**50-100**	**100-150**	**150-200**
BR	39.8%	41.1%	33.3%	0.0%
CPE	9.7%	21.5%	33.3%	20.0%
PP	6.5%	6.5%	12.5%	20.0%
PET	19.4%	7.5%	8.3%	20.0%
Flu	2.2%	9.3%	12.5%	0.0%
PE	1.1%	3.7%	0.0%	20.0%
PVC	0.5%	0.9%	0.0%	20.0%
PS	1.1%	0.9%	0.0%	0.0%
SBS	3.2%	3.7%	0.0%	0.0%
PU	1.1%	0.9%	0.0%	0.0%
EAA	12.4%	3.7%	0.0%	0.0%
CPI	1.6%	0.0%	0.0%	0.0%
EPN	0.5%	0.0%	0.0%	0.0%
ABS	0.5%	0.0%	0.0%	0.0%
PLA	0.5%	0.0%	0.0%	0.0%
Total MPs	100.0%	100.0%	100.0%	100.0%

Abbreviations: BR, butadiene rubber; CPE, chlorinated polyethylene; PP, polypropylene; PET, polyethylene terephthalate; Flu, fluororubber; PE, polyethylene; PVC, polyvinyl chloride; PS, polystyrene; SBS, styrene-butadiene-styrene; PU, polyurethane; EAA, ethylene acrylic acid; CPI, chlorinated polyisoprene; EPN, phenolic epoxy resin; ABS, acrylonitrile butadiene styrene; PLA, polylactic acid.
